# Gastrin mediates cardioprotection through angiogenesis after myocardial infarction by activating the HIF-1α/VEGF signalling pathway

**DOI:** 10.1038/s41598-021-95110-7

**Published:** 2021-08-04

**Authors:** Rui Wang, Ziyue Zhang, Zaicheng Xu, Na Wang, Dezhong Yang, Zhi Zhao Liu, Qiao Liao, Xuewei Xia, Caiyu Chen, Jialing Shou, Liangpeng Li, Wei Eric Wang, Chunyu Zeng, Tianyang Xia, Hongyong Wang

**Affiliations:** 1 Department of Cardiology, Chongqing Institute of Cardiology, Daping Hospital, Third Military Medical University, Chongqing, 400042 China; 2 Department of Military Medical Geography, Army Health Service Training Base, Third Military Medical University, Chongqing, 400038 China; 3 Institute of Health Biological Chemical Medication, Chongqing Institute of Green and Intelligent Technology, Chongqing, 400714 China

**Keywords:** Cardiology, Cardiovascular diseases

## Abstract

Acute myocardial infarction (MI) is one of the leading causes of death in humans. Our previous studies showed that gastrin alleviated acute myocardial ischaemia–reperfusion injury. We hypothesize that gastrin might protect against heart injury after MI by promoting angiogenesis. An MI model was simulated by ligating the anterior descending coronary artery in adult male C57BL/6J mice. Gastrin was administered twice daily by intraperitoneal injection for 2 weeks after MI. We found that gastrin reduced mortality, improved myocardial function with reduced infarct size and promoted angiogenesis. Gastrin increased HIF-1α and VEGF expression. Downregulation of HIF-1α expression by siRNA reduced the proliferation, migration and tube formation of human umbilical vein endothelial cells. These results indicate that gastrin restores cardiac function after MI by promoting angiogenesis via the HIF-1α/VEGF pathway.

## Introduction

Cardiovascular diseases, especially acute myocardial infarction (MI) and heart failure, are the number one cause of death globally and have rapidly increased in recent years^1^. The treatment of MI is based on two principles: short-term revascularization and long-term angiogenesis. Short-term revascularization, such as coronary artery bypass grafting or percutaneous coronary intervention, is the standard treatment and is currently widely used. However, the beneficial effect of revascularization is limited because the density of myocardial microvessels in the infarction border zone is low and the local perfusion is poor^2,3^. Therefore, additional long-term angiogenic therapeutic options are required.

The hypoxia-inducible factor 1 (HIF-1α)/vascular endothelial growth factor (VEGF) pathway is the main signalling pathway regulating long-term angiogenesis after MI^4^. HIF-1 is a transcriptional activator of VEGF, which is essential for initiating early cellular responses to hypoxia^5^. The HIF-1α/VEGF pathway is involved in myocardial remodelling and peri-infarction vascularization. Enhancement of the HIF-1α/VEGF pathway can prevent the expansion of the MI area and improve the survival rate of myocardial cells in the border zone of the MI area^6^. Promoting the HIF-1α/VEGF pathway is considered a feasible strategy to improve the prognosis of MI^7^.

Gastrin, originally identified as a peptide hormone that promotes digestion, is highly expressed in the heart and coronary arteries^8^. Previous studies have shown an elevated capillary density at the edge of colorectal adenocarcinoma in patients with hypergastrinemia^9^. The effect of gastrin on microangiogenesis was also observed in an in vivo model of glioblastoma^10^, suggesting that gastrin promotes angiogenesis. Our present study showed that gastrin promotes cardiac function after MI by promoting angiogenesis via the HIF-1α/VEGF pathway, which might provide an alternative therapeutic method for MI.

## Materials and methods

### Animals and reagents

Eight-week-old male C57BL/6 J mice (20–30 g, SPF Biotechnology, Beijing, China), obtained from the animal centre of the Third Military Medical University, were fed a normal mouse diet. All animal experiments were conducted in accordance with the Guide for the Care and Use of Laboratory Animals published by the US National Institutes of Health (NIH publication no. 85–23, revised 1996) and approved by the animal care and use committee of the Third Military Medical University (AMUWEC20181574). We confirm that the study was carried out in compliance with the ARRIVE guidelines and used an individual animal as the experimental unit.

### Establishment of the acute MI model and grouping

The species and number of models of MI were chosen according to a previous article^11^ and our past experience to guarantee the survival rate and to meet the needs of the experimental tests. Anaesthesia was administered by inhalation of isoflurane (Baxter, Guayama, US) throughout the operation. Normal breathing was maintained by connecting endotracheal intubation through a ventilator (Hugo Sachs Elektronik, March-Hugstetten, Germany), the respiratory rate was maintained at 100–120 times/min, and the tidal volume was 1 mL. Left thoracotomy was performed to expose the chest cavity, and the left anterior descending coronary artery (LAD) was ligated with 6–0 silk thread 2 mm below the lower margin of the left atrial appendage. The white colour of the front part of the left ventricle was used to define a successful model. For the mice in the sham operation group (n = 20), the ligature was not secured, and the thoracic cavity of all mice was sutured with 4–0 thread after surgery. All mice were subcutaneously (S.C.) injected with buprenorphine (0.5 mg/kg) every 6 h for two days after surgery. All mice scoring greater than 3/50 on the welfare scoring system were defined as ‘in pain’ and received an extra dose of 0.01 mg of buprenorphine S.C. The total number of mice that underwent MI surgery was 223 mice, and 63 of them died after surgery. Then, the remaining 160 mice were randomly divided into 4 groups by a random number generator: the MI + normal saline (NS) group (n = 40), MI + gastrin group (30 μg/kg/d, n = 40), MI + CI-988 group (300 μg/kg/d, n = 40) and MI + gastrin + CI-988 group (n = 40). Intraperitoneal injection of gastrin (Sigma, Saint Louis, US) and CI-988 (Tocris, Bristol, UK, CCKBR antagonist, which inhibits the effects of gastrin by blocking CCKBR) was performed twice daily for 2 weeks, and the animals in the MI + NS group and the sham group were injected with the same amount of normal saline daily. All mice were located on the same shelf in the animal centre, and the staff were required to randomly change the positions on the same shelf when the pads were changed every day. We administered intraperitoneal injections according to the order of the positions. Except for the mice that died during the operation or within 4 weeks after the operation, the experimental mice were in good condition and underwent the subsequent sampling and different experiments.

### Echocardiography

Cardiac function and structure were assessed using a Vivid 9 high-frequency colour Doppler ultrasound system (GE Healthcare, Boston, US) operated by a blinded and skilled technologist. In brief, isoflurane inhalation anaesthesia was implemented by an anaesthetic canister, and the left thoracic fur was carefully removed. Then, a two-dimensional echocardiogram measurement was performed, while left ventricular ejection fraction (LVEF), left ventricular fractional shortening (LVFS), left ventricular internal diameter in systole (LVIDs) and left ventricular internal diameter in diastole (LVIDd) were acquired in M-mode and B-mode echocardiograms from the LV parasternal long-axis view. The ultrasound probe was placed vertically on the left side of the mouse’s sternum at an angle of approximately 20–30 degrees with the long axis of the mouse’s body to obtain the parasternal left ventricular long-axis view. The long-axis view in B-mode is shown in Supplementary Figure S6.

### Detection of the MI size

The mice were sacrificed by intraperitoneal injection of 0.15 mL of 2.5% pentobarbital sodium (Sigma, Saint Louis, MI), and the hearts were removed immediately. After PBS rinses, 5–7 sections with a thickness of 2 mm were taken from the centre and upper and lower parts of the infarcted white area. Then, the sections were incubated in a water bath for 15–20 min with a premade 1% solution of 2,3,5-triphenyltetrazolium chloride (TTC, Saint Louis, US). After dyeing, the sections were fixed with 4% paraformaldehyde for 48 h and then photographed with a camera. The heart sections were taken with ImageJ (NIH, Bethesda, USA), and the infarct area, which was white, was calculated.

### Histopathology and immunofluorescence staining

The hearts of mice were fixed with 4% paraformaldehyde for 24 h, dehydrated, embedded in paraffin, and then sliced into 4 μm thick slices, followed by Masson tricolour staining (Solarbio, Beijing, China). Histopathological changes were observed after treatment. The sections were recorded by microscope imaging, the blue represents the fibrosis and the fibrotic area and normal area were calculated by ImageJ. Except for the samples with poor dehydration, damaged sections, or the sections with failed Masson’s trichrome stain, all samples were used for the statistical analysis.

Immunofluorescence staining was performed with anti-CD31 antibody (Abcam, Cambridge, UK), as described earlier^12,13^. In brief, the heart tissue (mainly part of the MI area) was fixed in 30% sucrose paraformaldehyde fixative solution for 24 h, and then, the samples were O.C.T (Sakura, Japan) embedded and sliced at -20–25℃. The slices were 4 μm thick. The sections were incubated with CD31 antibody at 4 °C overnight, rinsed with PBS 3 times and incubated with immunofluorescence secondary antibody (Cell Signaling Technology, Boston, USA) for 2 h. DAPI (Beyotime, Shanghai, China) was used to stain cells after PBS washes, and the slices were sealed with antifade mounting medium (Beyotime, China) and observed under a fluorescence microscope (Leica, Wetzlar, Germany). The red dots of CD31 immunofluorescence represent angiogenesis. Except for the samples with damage and the incomplete frozen sections or those with failed DAPI staining, all samples were used for statistical analysis.

### Cell culture and treatment

Human umbilical vein endothelial cells (HUVECs) were purchased from the American Type Culture Collection (ATCC, Rockville, MD). The cells were cultured in RPMI 1640 basic endothelial growth medium (Gibco, MA, USA) with 10% foetal bovine serum (Gibco, MA, USA) at 37 °C in 5% CO_2_. The medium was replaced every 2 days. Cells were treated with gastrin (10^–9^ mol/L) and/or CI-988 (10^–8^ mol/L) for 3 to 72 h, according to the experimental needs. In general, cell experiments were divided into at least 4 groups: control, gastrin, CI-988 and gastrin + CI-988. The details are as follows.

### Tube formation assay

In vitro angiogenesis was studied by the matrix gel method. HUVECs (1 × 10^6^ cells/injection) were incubated with VEGF, gastrin, CI-988, gastrin + CI-988 or serum-free medium for 3 h at 37 °C. Fifty microlitres of frozen growth factor-lowering matrix gel (BD Biosciences, Bedford, MA) was added to each well of 96-well plates and incubated for 30 min at 37 °C. Then, 50 μL of different pretreated HUVECs were inoculated in each well. The images were photographed after 6 h. ImageJ software was used to determine the total tube length.

### Cell proliferation assay

The viability of HUVECs was evaluated by using the CCK-8 assay (Biotechnology Company of Jiangsu Province, China). HUVECs were inoculated in 96-well plates at a density of 2 × 10^4^ cells/24 h, exposed to 10 μL of CCK-8 reagent with different treatments and incubated at 37 °C for an additional 4 h. The absorbance value at a wavelength of 450 nm was detected after incubation with an automatic reader (Tecan Group, Ltd., Mannedorf, Switzerland).

### Scratch test

The scratch experiment^14^ was performed in a 6-well plate. When the density of HUVECs reached 70%, a straight line was drawn along the centre of the plate with the sterilized pipette head, and the original culture was continued after the microscope examination. HUVECs were incubated with gastrin, CI-988, gastrin + CI-988 and serum-free medium. After 48 h, a micrograph was taken again, and the distance was measured from 6 or more fields to confirm the migration level.

### Transwell migration assay

Cell migration was analysed using a Transwell migration assay. In short, cells were plated in the top well of the Transwell chamber. After incubation for 24 h, the nonmigrating cells were removed with cotton swabs, and the migrated cells under the membrane were stained with crystal violet.

### Downregulation of HIF-1α expression in HUVECs

To downregulate the expression of HIF-1α in HUVECs, we used siRNA for cell transfection, and Lipofectamine 2000 was used to improve the interference efficiency. In detail, we plated cells to 70% confluence at the time of transfection. Then, Lipofectamine 2000 Reagent (Invitrogen, California, USA) or HIF-1α siRNA (5'-GGAACATGATGGTTCACTT-3', RiboBio, Guangdong, China) was premixed and incubated with Opti-MEM (Gibco, MA, USA) for 10 min and incubated with both mixtures for 5 min (in 6-well plates, 5 μL of Lipofectamine 2000 Reagent, 5 μL of HIF-1α siRNA and 300 μL of Opti-MEM in each well). The medium on the plate was removed and washed with PBS twice. Finally, medium with no serum and a siRNA mixture were added for incubation. The medium was removed 6 h after transfection and replaced with fresh medium containing 10% serum.

### Western blotting

Total proteins were isolated from the left ventricular tissue and HUVECs according to previous studies^15^. Then, the protein concentration was determined by the bicinchoninic acid (BCA) method, the isometric protein marker was added, and the protein was denatured at 100 °C for 5 min. The protein separated on the polyacrylamide gel was transferred to a nitrocellulose filter membrane, and the resulting membrane was sealed in 5% skim milk at room temperature for 1 h and then incubated with the corresponding antibodies, including mouse anti-HIF-1α monoclonal antibody (28b) (Santa Cruz Biotechnology, CA, USA, 1:200). After incubation at 4℃ overnight, the membrane was incubated with fluorescent-conjugated secondary antibodies (Invitrogen, California, USA, 1:2000) for 1 h at room temperature. The signal was detected using chemiluminescence with an image analyser. ImageJ (NIH, Bethesda, USA) was used to analyse the average grey value of each band.

### Real-time quantitative polymerase chain reaction

mRNA levels of HIF-1α and VEGF were quantified by quantitative polymerase chain reaction (qPCR) with the following specific primers: (1) For human beings, HIF-1α, forward (5'-GTCTGAGGGGACAGGAGGAT-3') and reverse (5'-CTCCTC AGGTGGCTTGTCAG-3'); VEGF, forward (5'-AGGGCAGAATCATCACGAAGT -3') and reverse (5'-AGGGTCTCGATTGGATGGCA-3'); GAPDH forward (5'-AGA AGGCTGGGGCTCATTTG-3') and reverse (5'-AGGGGCCATCCACAGTCTTC-3'). (2) For mice, HIF-1α, forward (5'-CGCCTCTGGACTTGTCTCTT-3') and reverse (5'-TCGACGTTCAGAACTCATCCT-3'); VEGF, forward (5'-TATTCAGCGGACT CACCAGC-3') and reverse (5'-AACCAACCTCCTCAAACCGT-3'); and GAPDH forward (5'-ACGGCAAATTCAACGGCACAG-3') and reverse (5'-AGACTCCACGAC ATACTCAGCAC-3').

RNAiso Plus reagent (TaKaRa, Tokyo, Japan) was used for total RNA extraction, and according to the manufacturer’s instructions, cDNA synthesis was performed using PrimeScript RT Master Mix (TaKaRa, Tokyo, Japan). For qPCR, the mRNA levels of HIF-1α, VEGF and GAPDH were measured by using TB Green Premix Ex Taq (TaKaRa, Tokyo, Japan) and the Thermal Cycler Dice Real-Time System (Bio-Rad, Hercules, CA, United States). The following PCR conditions were applied: 95 °C for 3 min, 40 cycles at 95 °C for 10 s and 62 °C for 30 s followed by 62 °C for 10 s. The relative mRNA expression levels were evaluated using the 2 − ΔΔCt method and normalized to that of GAPDH.

### VEGF assay

The amount of VEGF produced by HUVECs was determined by a Human VEGF SimpleStep ELISA Kit (Abcam, Cambridge, UK). HUVECs were cultured in a 24-well plate in the presence of gastrin (10 − 9 M) with or without CI-988 (10^−5^ M), and we used PBS as a control. VEGF released from HUVECs was measured with a VEGF SimpleStep ELISA Kit, according to the manufacturer’s suggestion.

### Statistical analysis

**Figure 1:**
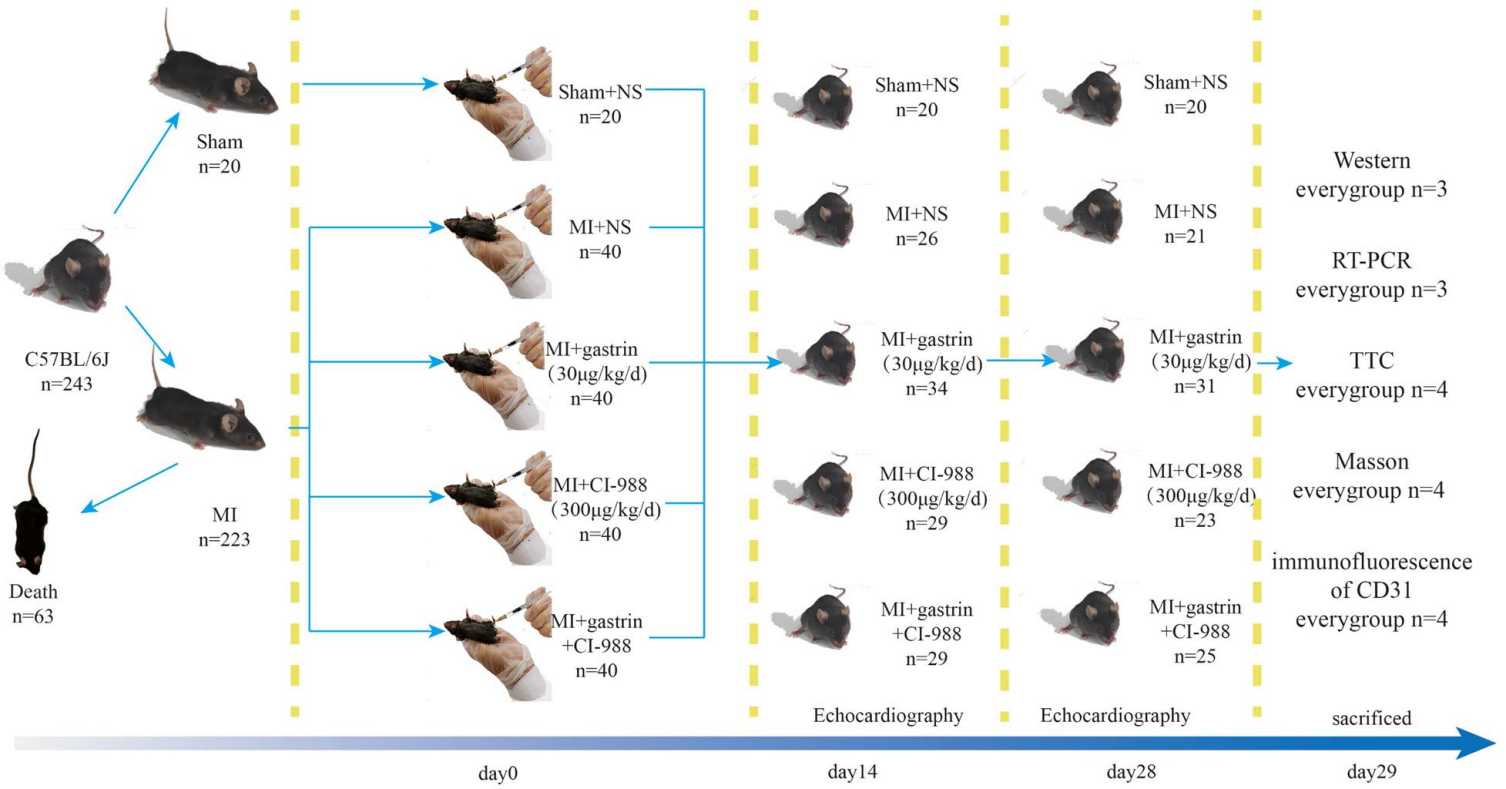
Flow chart of animal Experiments

All data are represented as the mean ± SD. Differences between multiple groups were analysed by ANOVA. We carefully reviewed all the statistical analyses, and all the data analyses in the bar charts were performed by one-way ANOVA for all and LSD for pair comparisons. Homogeneity of variance was detected before the analysis (P > 0.05). Whenever assumptions of normality and homogeneity of variance were questionable, we used Mann–Whitney analysis. The survival rate is presented as a Kaplan–Meier curve and was compared by the log-rank test. *P* ≤ 0.05 was defined as statistically significant. SPSS 20.0 statistical software was used for all statistical analyses, and Pass15.0 was used to calculate the sample size of the animal survival experiment (Fig. 1).

### Ethical approval

All applicable international, national, and/or institutional guidelines for the care and use of animals were followed. The protocol (including the research question, key design features, and analysis plan) was prepared before the study and registered at the animal care and use committee of the Third Military Medical University. The data were kept in the Department of Cardiology, Chongqing Institute of Cardiology, Daping Hospital, Third Military Medical University.

## Results

### Gastrin improves cardiac function after MI

**Figure 2:**
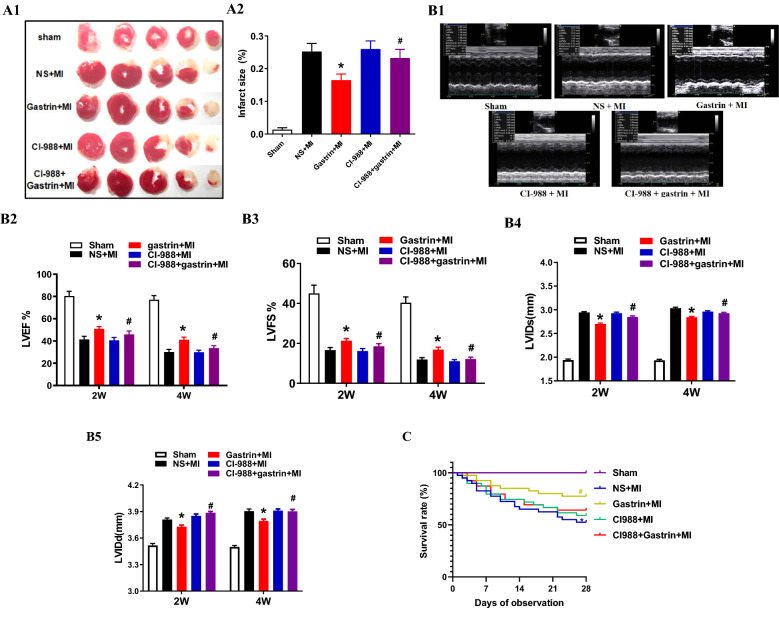
Gastrin improved the prognosis of myocardial infarction (MI). (**A**) The infarcted region was assessed based on TTC staining of heart sections, and the infarcted field was white. The MI + gastrin group showed a decreased infarcted area (*P < 0.05 vs. no gastrin, #*P* < 0.05 vs. the gastrin only group, n = 4). (**B1**) Representative M-mode images through echocardiography after acute MI for 2 and 4 weeks. Left ventricular wall motion in the MI + gastrin group was improved after AMI for 4 weeks. The corresponding B-mode images are presented in Supplementary Fig. 6 (Figure S6). (**B2**) Left ventricular ejection fraction (LVEF), (**B3**) Left ventricular fractional shortening (LVFS), (**B4**) left ventricular internal diameter in systole (LVIDs) and (**B5**) left ventricular internal diameter in diastole (LVIDd) were measured by echocardiography and were improved after treatment with gastrin (*P < 0.05 vs. no gastrin, #*P* < 0.05 vs. the gastrin only group, n = 10). (**C**) Survival charts for all groups of mice at 4 weeks. The survival rate was significantly improved in the MI + gastrin group (*P < 0.05 vs. sham, #*P* < 0.05 vs. no gastrin).

We demonstrated the protective effect of gastrin on C57BL/6 J mice by intraperitoneal injection of gastrin (30 μg/kg/d) for 2 weeks after MI. The infarct size and cardiac function were evaluated at 2 and 4 weeks post-MI. Compared with the control mice, the gastrin-treated mice had a smaller infarct size (Fig. 2A) and increased cardiac function, as determined by the increasedLVEF, LVFS, LVIDd and LVIDs values (Fig. 2B) post-MI. The survival rates of the gastrin-treated mice were higher than those of the control mice with MI (Fig. 2C). Moreover, CI-988, a cholecystokinin receptor inhibitor, partially blocked the protective effect of gastrin on MI.

**Figure 3:**
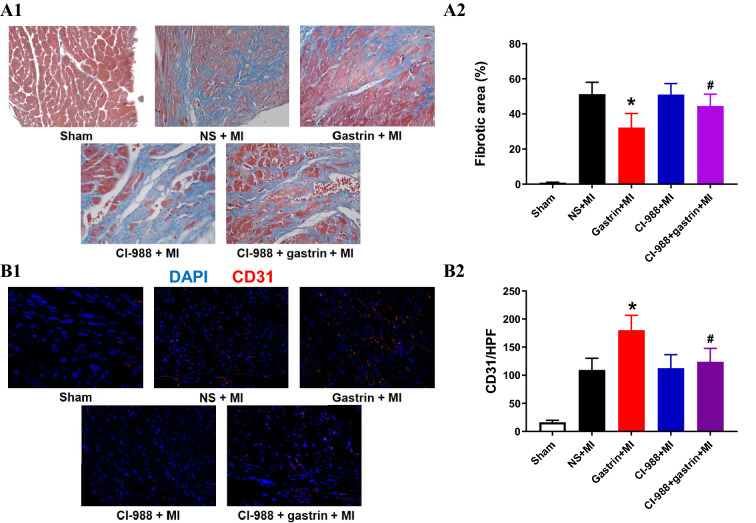
Gastrin optimized myocardial remodelling after MI. (**A1**) Representative Masson’s trichrome images of left ventricle (LV) sections in all groups showed decreased numbers of fibroblasts (blue cells) in the infarcted hearts treated with gastrin. (**A2**) The fibrotic area was significantly decreased by gastrin injection (*P < 0.05 vs. no gastrin, #*P* < 0.05 vs. the gastrin only group, n = 4). (**B1**) Immunofluorescent staining of CD31 was performed to detect angiogenesis levels in infarcted hearts 1 month post-MI. (**B2**) The comparison of CD31^+^ cells per area (10,000 μm^2^) among different groups of infarcted hearts (*P < 0.05 vs. no gastrin, #*P* < 0.05 vs. the gastrin only group, n = 4).

In addition to the infarct size, we also assessed ventricular remodelling, including myocardial fibrosis and angiogenesis. Masson's trichrome staining showed that gastrin treatment decreased myocardial fibrosis (Fig. 3A); moreover, angiogenesis was increased by gastrin, as indicated by CD31 staining (Fig. 3B). These protective effects were also suppressed in the presence of CI-988.

### The increased expression of HIF-1α and VEGF by gastrin treatment in the heart after MI

**Figure 4:**
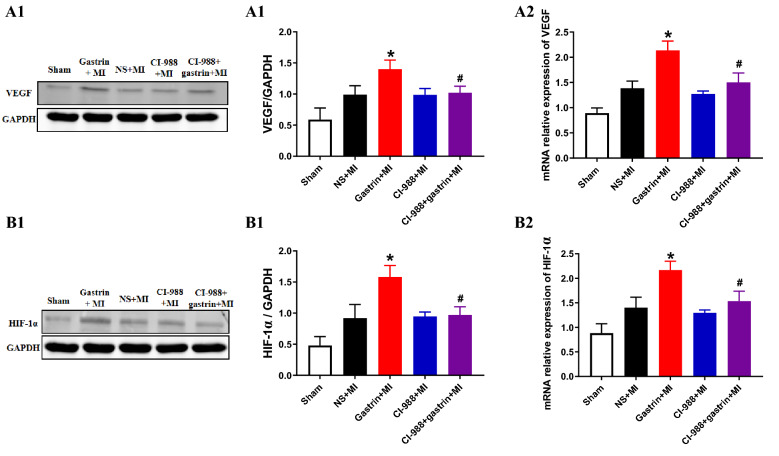
Gastrin increased the expression levels of HIF-1α and VEGF in the LV after MI for 4 weeks. The protein and mRNA expression levels of HIF-1a (**B1** and **B2**) and VEGF (**A1** and **A2**) were measured by immunoblotting and real-time PCR (*P < 0.05 vs. no gastrin, #*P* < 0.05 vs. the gastrin only group, n = 3). Full-length gels are presented in Supplementary Figs. 1—3 (Figures S1 – S3).

Due to the importance of VEGF in angiogenesis, we assessed its expression in hearts after MI. MI increased VEGF expression, and gastrin further enhanced VEGF expression in the hearts after MI (Fig. 4A). As a factor upstream of VEGF, HIF-1α expression was increased further by gastrin treatment (Fig. 4B), as confirmed by Western blotting and qPCR. In the presence of CI-988, the stimulatory effects of gastrin on both VEGF and HIF-1α expression were blocked, indicating that the stimulatory effect occurred via the gastrin receptor (which shares the same receptor with cholecystokinin).

### Gastrin promotes tube formation by activating HIF-1α

**Figure 5:**
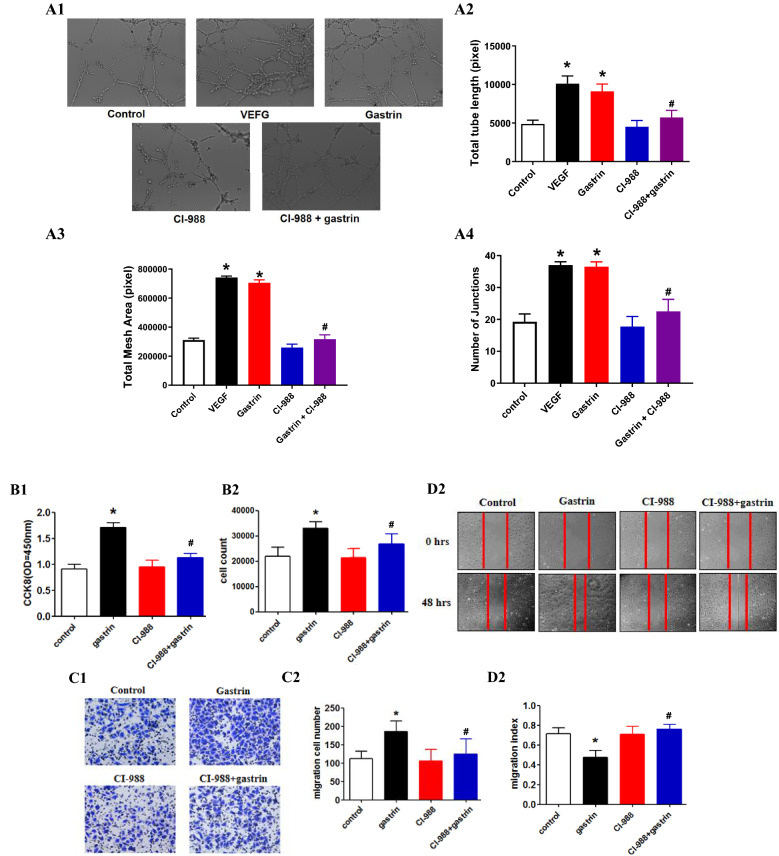
Effects of gastrin on capillary tube formation, proliferation and migration. HUVECs were seeded in Matrigel-coated wells in different groups. (**A1**) Representative photomicrograph of a tubular experiment, which showed an increase in tube formation in the gastrin group. Total tube length (**A2**), total mesh area (**A3**) and number of junctions (**A4**) were determined to represent the level of tube formation (*P < 0.05 vs. the control, #*P* < 0.05 vs. the gastrin only group, n = 4. (**B**) The absorbance and the number of cells were measured with the CCK-8 kit, which showed that gastrin increased the proliferation of HUVECs (*P < 0.05 vs. control, #*P* < 0.05 vs. the gastrin only group, n = 6). (**C**) Effects of gastrin and/or CI-988 on the Transwell migration of HUVECs and (D) increasing wound-healing in HUVECs (*P < 0.05 vs. the control, #*P* < 0.05 vs. the gastrin only group).

To demonstrate the contribution of gastrin to angiogenesis, we studied its effect on HUVECs. Gastrin significantly promoted capillary-like tube formation and angiogenesis (Fig. 5A), as evaluated by the number of junctions, total tube length and total mesh area. Furthermore, the results of the CCK-8 assay showed that gastrin significantly increased HUVEC proliferation compared with the control (Fig. 5B). In addition, gastrin significantly increased the migration of HUVECs, as confirmed by Transwell assays and wound-healing assays (Fig. 5C, D).

**Figure 6:**
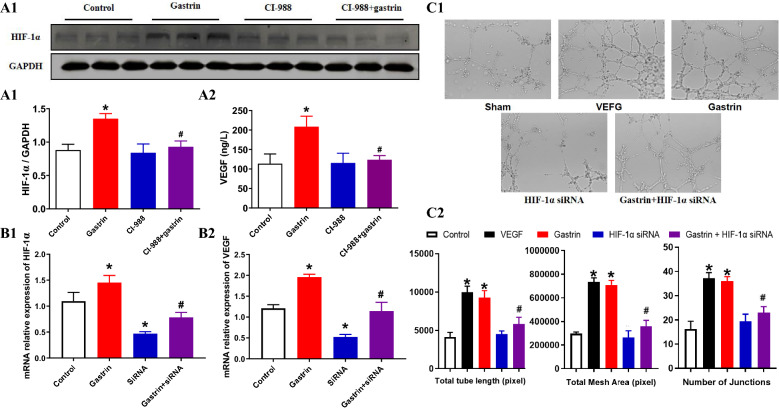
Identification of the pathway after siRNA treatment in vitro. (**A1**) Gastrin increased the mRNA transcription of HIF-1α in vitro, and CI-988 effectively reduced the transcription levels of HIF-1α. (**A2**) Gastrin also upregulated the VEGF content in the cell supernatant (*P < 0.05 vs. the control, #*P* < 0.05 vs. the gastrin only group, n = 8). (**B**) Through the knockdown of HIF-1α by siRNA, it was found that the regulatory effect of gastrin on VEGF was blocked (*P < 0.05 vs. the control, #*P* < 0.05 vs. the gastrin only group, n = 3). Full-length gels are presented in Supplementary Figs. 4 and 5 (Figures S4 and S5). (**C**) HIF-1α knockdown inhibited the tube formation of HUVECs after treatment with gastrin. Total tube length, total mesh area and number of junctions were determined to represent the level of tube formation (*P < 0.05 vs. the control, #*P* < 0.05 vs. the gastrin only group, n = 4).

Consistent with the in vivo experiment, we found that gastrin treatment increased VEGF and HIF-1α expression (Fig. 6A). Downregulation of HIF-1α expression by siRNA blocked the upregulation of VEGF expression by gastrin, indicating that gastrin regulates the expression and secretion of VEGF through HIF-1α (Fig. 6B). Moreover, inhibition of HIF-1α by siRNA blocked the stimulatory effect of gastrin on tube formation in HUVEC tubular experiments (Fig. 6C).

## Discussion

In recent years, the gastrointestinal hormone gastrin, via its receptor cholecystokinin B receptor (CCKBR or CCK2R), has been found to exert beneficial cardiovascular effects, such as improving cardiac function. Clinical studies have shown that patients with MI have lower infarct sizes during mealtime, indicating that gastrointestinal hormones, including gastrin, may play a vital role in the protection against MI^16,17^. Experiments in animals such as pigs and mice have also found protective effects of gastrin on cardiac function after MI^18,19^. Blood supply, one of the main factors affecting cardiac function, is ensured by timely revascularization and adequate angiogenesis during MI^20^. Studies have shown the effect of gastrin on revascularization. For example, in experimental pigs, gastrin increased coronary blood flow and cardiac function through the activation of CCK receptors, β-adrenoceptors and nitric oxide (NO) release^18^. In our previous study, we also found a protective effect of gastrin on myocardial ischaemia–reperfusion after MI through both the SAFE and RISK pathways^19^. However, the effect of gastrin on angiogenesis is still unknown.

Studies have shown that gastrin promotes angiogenesis in many physiological and pathological tissues. In the digestive tract, gastrin promotes angiogenesis in gastric cancer tissues^21^ and enhances angiogenesis around ulcers to promote healing^22^. Other cancers, such as glioblastoma, can also be affected by gastrin by promoting angiogenesis^10^. Some factors, including hypoxia and cobalt irradiation, stimulate the secretion of gastrin, which promotes angiogenesis in hypoxic tissues, as well as melatonin^14–24^. However, the effects of gastrin on angiogenesis after MI have not been reported. In our present study, gastrin was shown to stimulate angiogenesis in the infarction border zone after MI in vivo; gastrin promoted the angiogenesis, proliferation and migration of HUVECs, indicating the regulatory effects of gastrin on angiogenesis after MI.

Although the mechanism of angiogenesis is still unclear, some factors, including VEGF, FGF, and hepatocyte growth factor, are critical in promoting angiogenesis after MI. HIF-1α is an important transcription factor regulating the cellular response to hypoxia and is one of the first adaptations of the human myocardium to ischaemia^4,5^. HIF-1α expression is continuously elevated in some key cells, such as myocardial and endothelial cells, at least 3 weeks after MI^25^. As a promoter of angiogenesis, HIF-1α modulates a wide range of targets, such as VEGF and stromal cell-derived factor 1, consequently promoting angiogenesis^26^. For example, in gastric cancer, high expression of gastrin increases HIF-1α/VEGF expression and promotes tumour angiogenesis. However, the effect of gastrin on the HIF-1α/VEGF pathway in MI is still unclear. In our study, exogenous administration of gastrin after MI in mice increased HIF-1α/VEGF expression in heart tissue, thereby promoting angiogenesis in the infarction border zone.

In conclusion, our study suggests that exogenous administration of gastrin protects cardiac function after MI by promoting angiogenesis; HIF-1α/VEGF might be involved in the underlying mechanisms. Exogenous gastrin administration can be a potential treatment to protect cardiac function after MI by promoting angiogenesis.

## Supplementary Information

Supplementary Figures.
