# Supplementary material for: Gastrin mediates cardioprotection through angiogenesis after myocardial infarction by activating the HIF-1α/VEGF signalling pathway
Source: Sci Rep. 2021 Aug 4;11:15836. doi: 10.1038/s41598-021-95110-7 (PMC8339006; doi:10.1038/s41598-021-95110-7)
Supplement: Supplementary file 1 — Supplementary Figures. [file 41598_2021_95110_MOESM1_ESM.pdf]

Supplementary Figure

Figure S1: Original full-length gels of VEGF expression in Figure 4A1

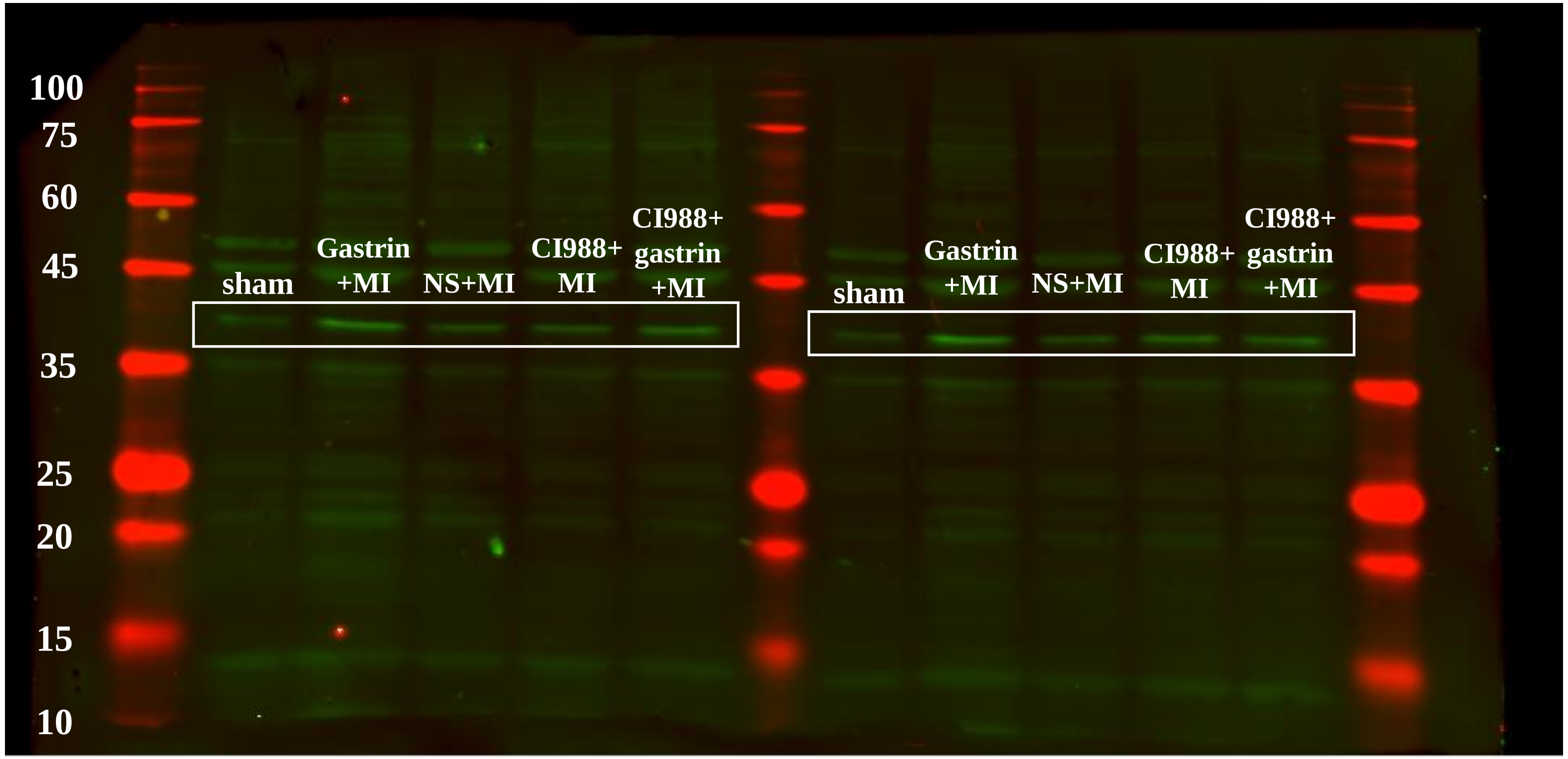

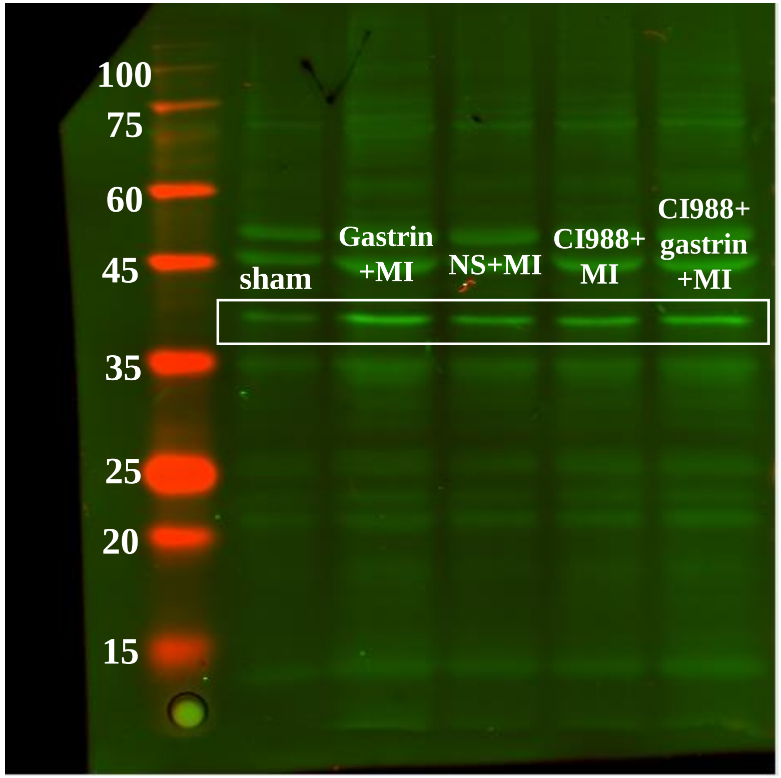

Figure S2: Original full-length gels of HIF-1α expression in Figure 4B1

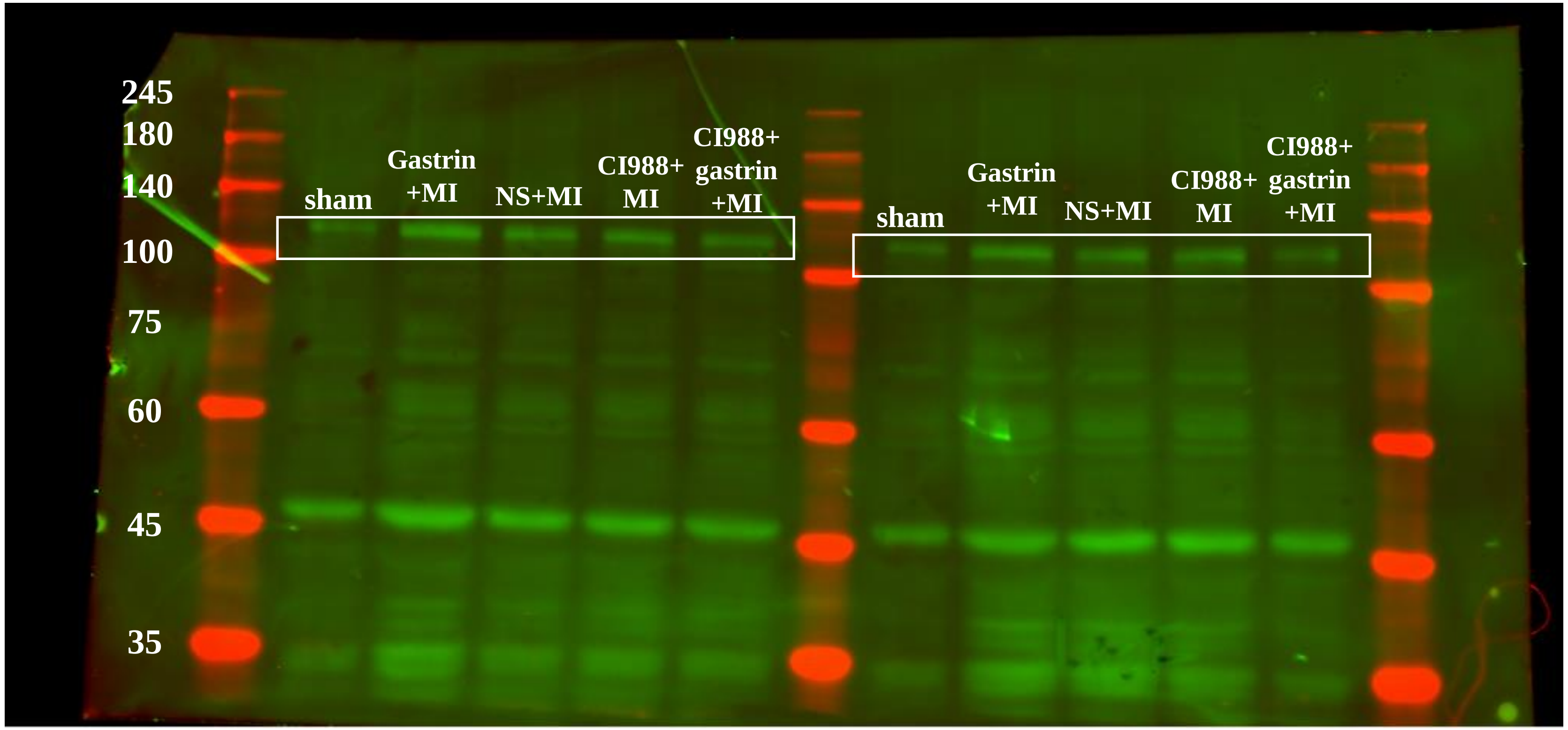

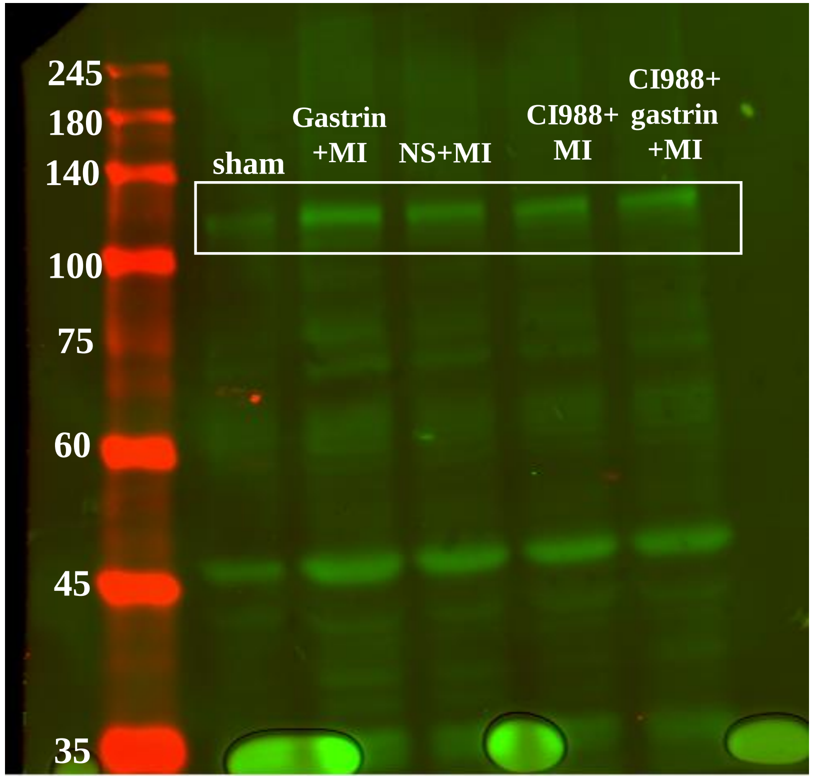

Figure S3: Original full-length gels of GAPDH expression in Figure 4A1 and 4B1 (used the same samples)

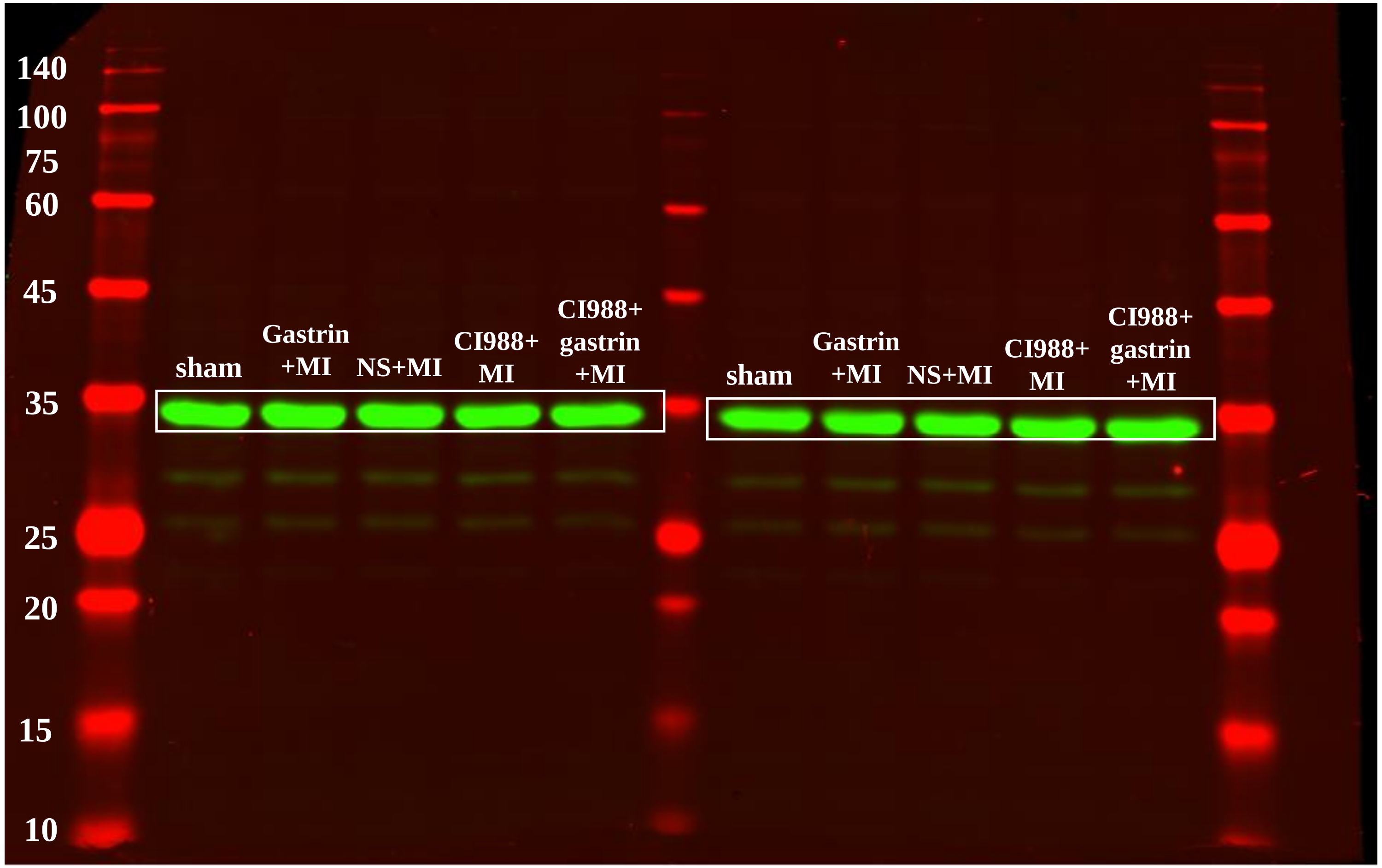

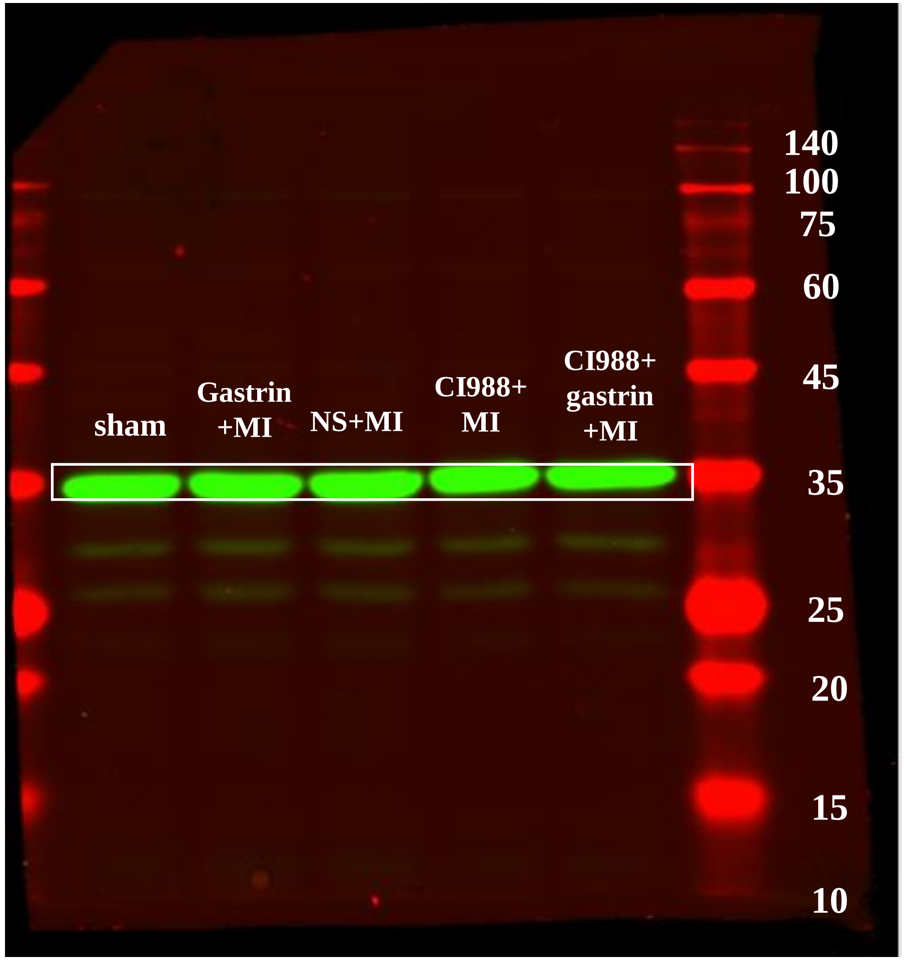

Figure S4: Original full-length gels of HIF-1α expression in Figure 6A1

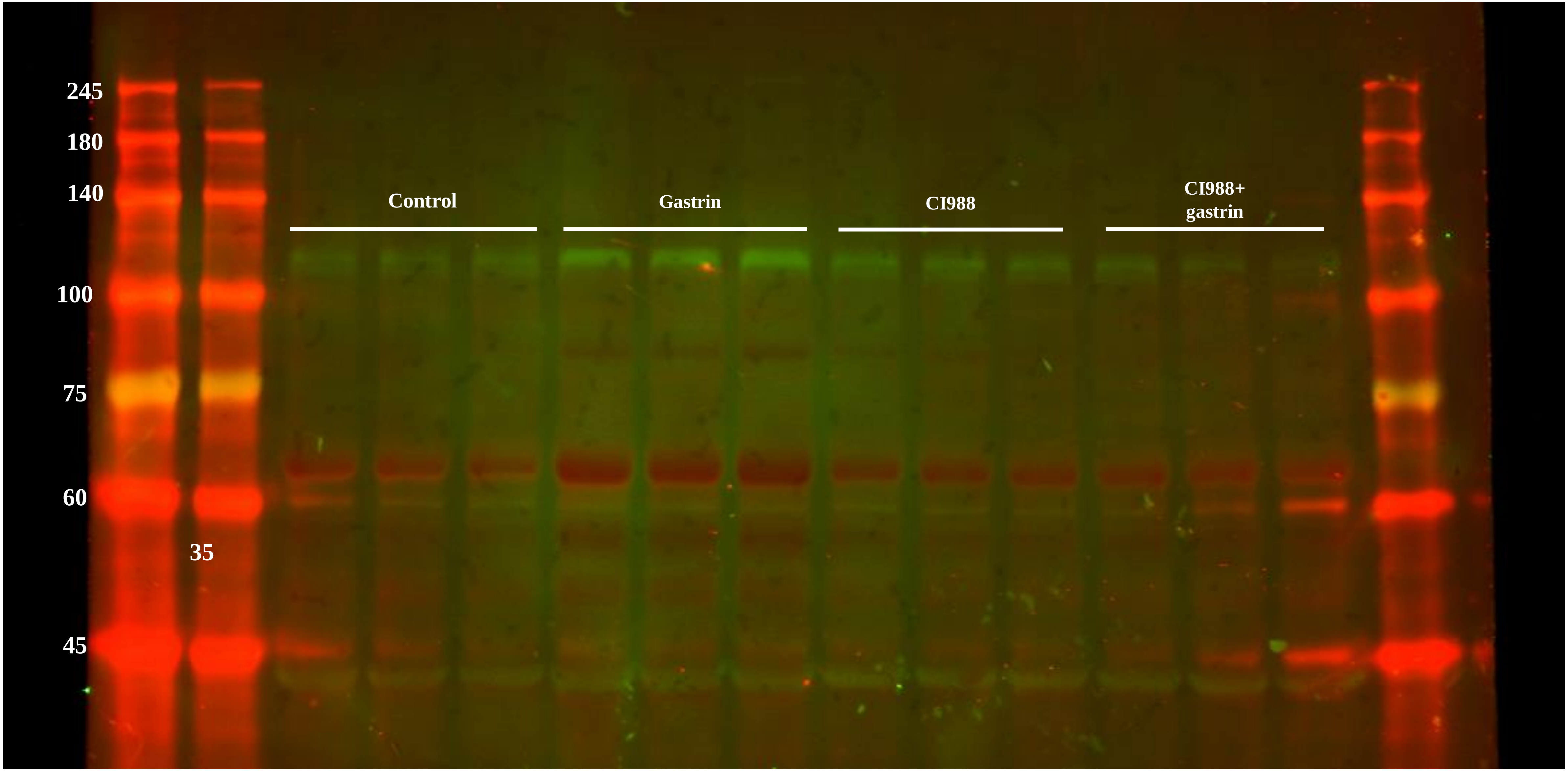

Figure S5: Original full-length gels of GAPDH expression in Figure 6A1 (used the same samples)

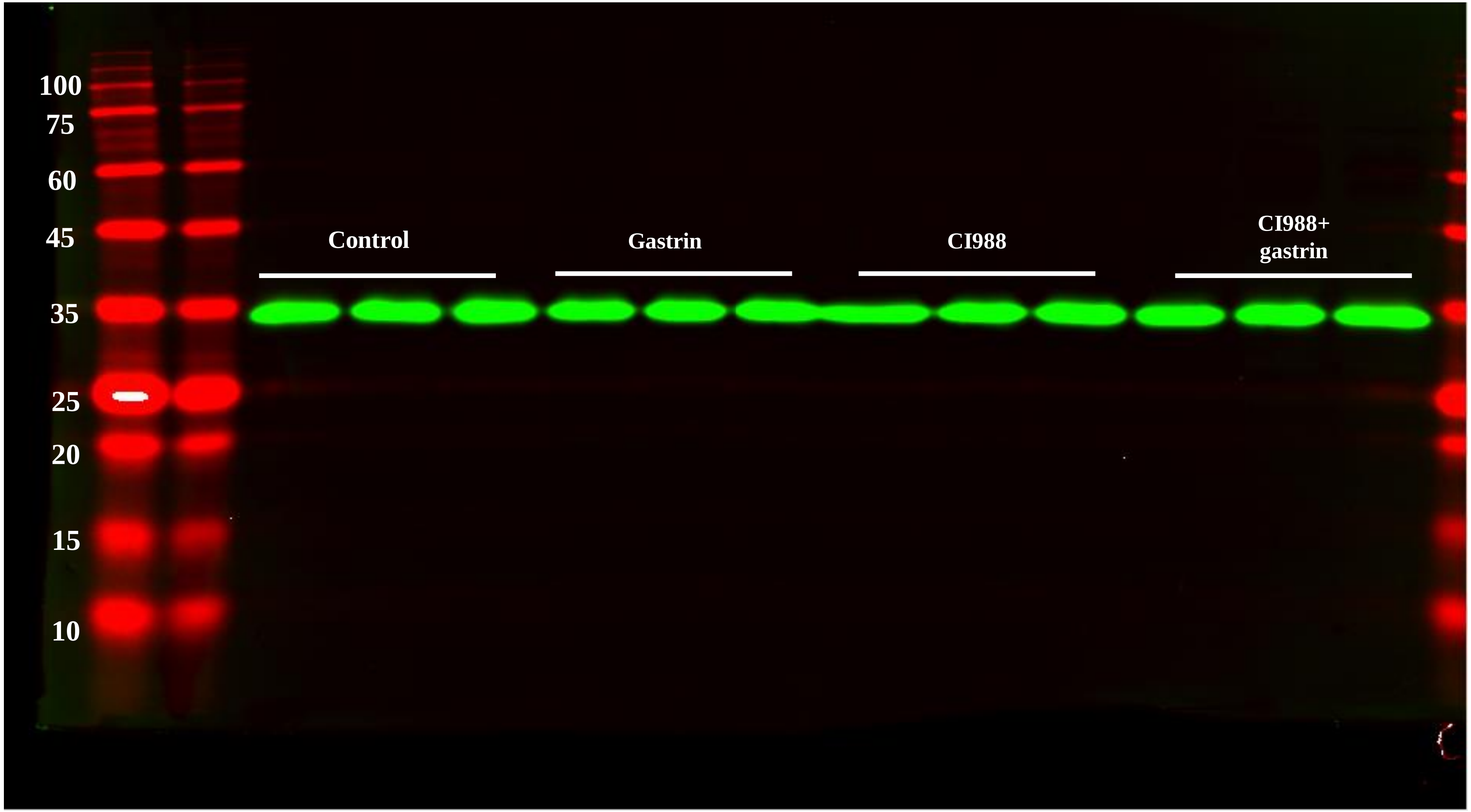

Figure S6: Original B-mode graph of mice hearts in Figure 2B1 (Corresponding mice)

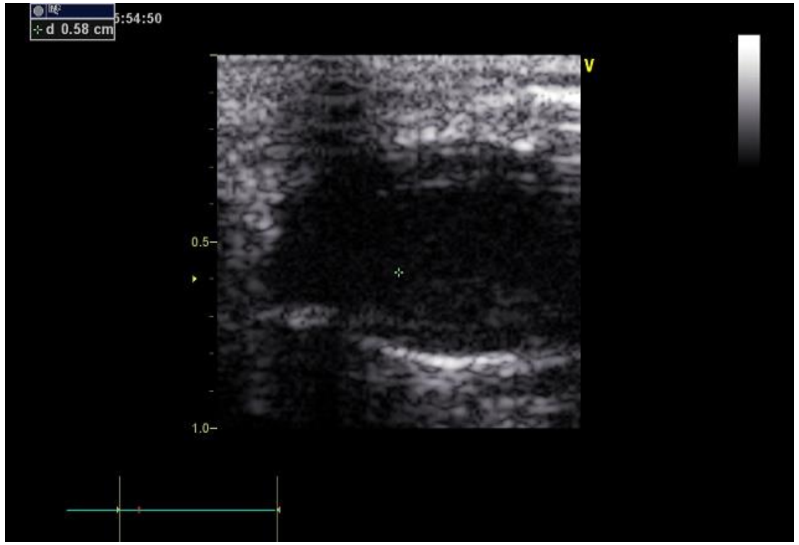

sham

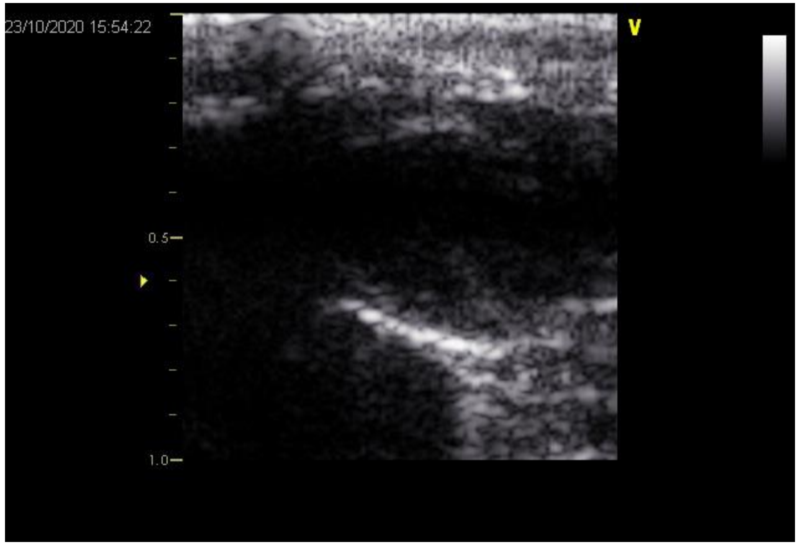

NS + MI

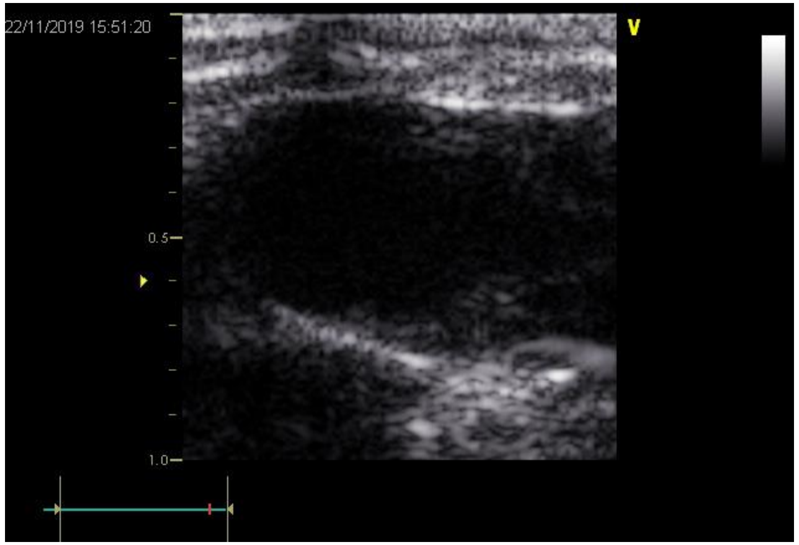

Gastrin + MI

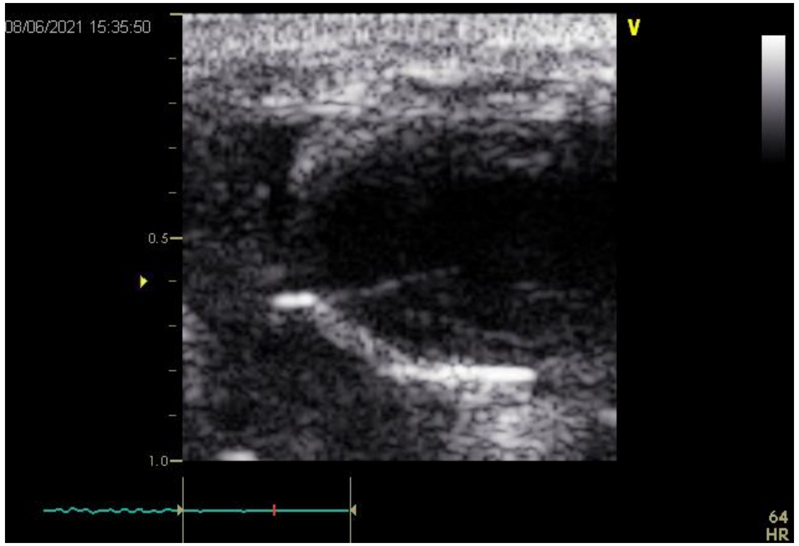

CI988 + MI

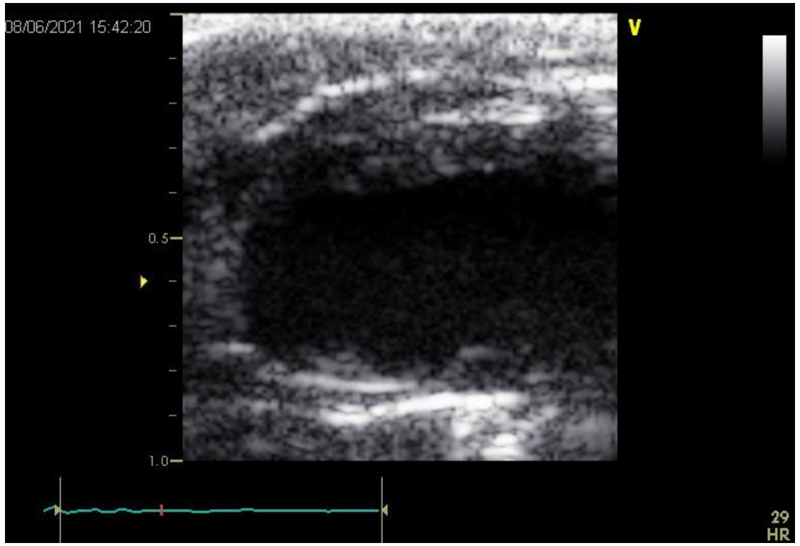

CI988 + gastrin + MI
